# Supplementary material for: Prevalence and Determinants of Excessive Screen Viewing Time in Children Aged 3–15 Years and Its Effects on Physical Activity, Sleep, Eye Symptoms and Headache
Source: Int J Environ Res Public Health. 2023 Feb 16;20(4):3449. doi: 10.3390/ijerph20043449 (PMC9958787; doi:10.3390/ijerph20043449)
Supplement: Supplementary file 1 [file ijerph-20-03449-s001.zip › ijerph-2076673-supplementary.pdf]

Questionnaire: Screen time

School **Name**______________	Child enrolment number (Not be filled by parent) __________

Child name __________________ Gender ___________ Age _________ Class ___________

1. 1 Socio-Demographic Information:

1.1.1 Name of Mother : ____________________

1.1.2 Educational Criteria of Responder

01: Uneducated
02: Elementary school
03: High school
04: High Sec
05: Graduate
06 Post Graduate

1.1.3 Family information

1.1.3.1 Type of Family ☐

01 Nuclear

02 Joint

1.1.3.2 Number of family members ....

1.1.3.3 Number of sibling ....

1.1.3.4 Age of sibling 1st………. 2nd……….. 3……… 4th …….

1.1.3.5 Overcrowding ☐

01 Yes

02 No

CRITERIA – no. of family member for room

1 Room 2 person

2 Room 3 person

3 Room 5 person

4 Room 7 person

5 Or more rooms 10 person (Additional 2 for each room)

1.1.3.6 Maternal information

1.1.3.6.1 Mother still living ☐

01 Yes
02 No

1.1.3.6.2 Mother age ………yrs

1.1.3.6.3 Mother's education

01: Uneducated
02: Elementary school
03: High school
04: High Sec
05: Graduate
06 Post Graduate

1.1.3.6.4 Mother's occupation

01 Housewife
02 Self employed
03 Farm-worker
04 Labour
05 Official job

1.1.3.7 Father's information.

1.1.3.7.1 Father still living

01 Yes
02 No

1.1.3.7.2 Father age ………yrs

1.1.3.7.3 Father's education

01: Uneducated
02: Elementary school
03: High school
04: High Sec
05: Graduate
06: Post Graduate

1.1.3.7.4 Father's occupation

01 Unemployed
02 Self employed
03 Farm-worker
04 Labour
05 Official Job

1.1.4 Socioeconomic status:

1.1.4.1 Place of residence

01 Urban
02 Rural

2. Screen viewing related questionnaire

2.1 Parent screen view time

	Please inform for how many hours/day you use TV, mobiles, tablet and computer/laptops in below mentioned categories							
	During week days				During week ends			
	For education	For entertain-ment	To keep yourself occupied	Others ______	For education	For entertain -ment	To keep yourself occupied	Others ______
TV	______							
Mobile								
Computer / Laptop								

2.2 Child screen view time

	Please inform for how many hours/day does your child use TV, mobiles, tablet and computer/laptops in below mentioned categories							
	During week days				During week ends			
	For education	For entertain-ment	To keep himself occupied	Others _____	For education	For entertain-ment	To keep himself occupied	Others ______
TV	______							
Mobile								
Computer / Laptop								

2.3 Do you place any restrictions on the amount of time your child can be in front of a screen (TV,mobile,tablet,computer/laptop)?

1. Yes 2. No

2.4 Do you place any restrictions on the content your child view on the screen(TV, mobile tablet,computer/laptop)? (movie ratings, YouTube video lyrics, parental settings, etc.)

1. Yes 2. No

2.5 Does your child interact with an electronic device soon after waking up?

1.Yes

a.as soon as he/she gets up b.within …….hours after waking up

2.No

2.6 Do you have TV in your bedroom?

1. Yes 2. No

2.7 Does your child use mobile phone at bed time?

1. Yes 2. No

2.8 Has your child ever complained of headache?

1. Yes 2. No

If yes: Do you think the headache can be because of using screen (any screen TV/Mobile/Tablet/Laptop/computer)

1. Yes 2. No

2.9 Has your child ever complained of eye pain?

1. Yes 2. No

If yes:Do you think the eye pain can be because of using screen (any screen TV/Mobile/ Tablet/Laptop/computer)

1. Yes 2. No

2.10 Has your child ever complained of itching in eyes?

1. Yes 2. No

If yes:Do you think the itching in eyes can be because of using screen (any screen TV/Mobile/ Tablet/Laptop/computer)

1. Yes 2. No

2.11 Do you think your child is habituated to screen (any screen TV/Mobile/ Tablet/Laptop/computer)

1. Yes 2. No

2.12 Does your child requires a screen (any screen TV/Mobile/ Tablet/Laptop/computer) for eating (lunch/breakfast/dinner)?

1. Yes 2. No

2.13 Does your child watch screen (any screen TV/Mobile/ Tablet/Laptop/computer) while eating (lunch/breakfast/dinner)?

1. Yes : ☐Breakfast ☐Lunch ☐Dinner

2. No

2.14 Do you know what is the maximum recommended time for viewing screen?

1. Yes 2. No

2.15 Does your child plays outside home?

1. Yes: How many minutes or hours/ day…………2.No

2.16 Does your child reads any other book apart from school book (story book/news paper etc)?

1.Yes: How much time-____ minutes or hours/ day

2.No

2.17 Do you/any other family member read/tell stories to the child

1. Yes: How frequently -☐Everyday ☐Once a week ☐Once a month

2.No

2.18 At what approximate time does your child goes to sleep at night every day?

2.19 How many hours does your child sleep at night?

______ Hrs in night

2.20 Do you think this survey was useful for your child?

1. Yes 2.No

2.21 What will you do now after knowing all this information?

I would like you to give information regarding screen time use-

According to American Academy Of Pediatrics; For children 18 months to 5 years, the AAP now recommends aiming for one hour of screen entertainment per day, rather than two.

AAP's new policy statement for children ages 5 to 17 actually has no standard **screen-time limit.**

Eight to 10 hours of sleep, time for homework, family time, and time that is completely screen-free. Look at the time that's left and seems reasonable for media use.

Content Validity Ratio of SVT questionnaire

Question no	Experts 1	Experts 2	Experts 3	CVR (NE − N/2) / (N/2)
2.1	√	×	√	0.33
2.2	√	√	√	1
2.3	√	√	√	1
2.4	√	√	×	0.33
2.5	√	√	√	1
2.6	√	√	√	1
2.7	√	√	√	1
2.8	√	√	√	1
2.9	√	√	√	1
2.10	√	√	√	1
2.11	√	×	√	0.33
2.12	√	√	√	1
2.13	√	√	√	1
2.14	√	√	√	1
2.15	√	√	√	1
2.16	√	√	×	0.33
2.17	√	√	√	1
2.18	√	√	√	1
2.19	√	√	√	1
2.20	√	√	√	1
				17.33

Content Validity Ratio = (NE − N/2) / (N/2)

- **NE** = Number of SME panelists indicating "essential"
- **N** = Total number of SME panelists

CVR = (0.33+1+1+0.33+1+1+1+1+1+1+0.33+1+1+1+1+0.33+1+1+1+1)

Total CVR = 17.33

Content Validity Index = (CVR) / Total number of questions

CVI = (0.33+1+1+0.33+1+1+1+1+1+1+0.33+1+1+1+1+0.33+1+1+1+1) / 20

CVI = 0.867
